# Comparison of Respiratory function recovery after esophagectomy: a retrospective analysis of surgical approaches

**DOI:** 10.1007/s10388-026-01241-z

**Published:** 2026-09-20

**Authors:** Naohito Sai, Kazushi Miyata, Takahiro Imaizumi, Yasukazu Kanie, Yuki Murata, Shizuki Sugita, Atsushi Ogura, Tomoki Ebata

**Affiliations:** 1 Division of Surgical Oncology, Department of Surgery, Nagoya University Graduate School of Medicine, 65 Tsurumai-cho, Showa-ku, Nagoya, 466-8550 Japan; 2 Department of Clinical Research Education, Nagoya University Graduate School of Medicine, Nagoya, Japan

## Abstract

**Background:**

Esophagectomy for patients with esophageal cancer is a highly invasive procedure that can lead to substantial postoperative pulmonary impairment. Minimally invasive approaches, such as video-assisted thoracoscopic surgery (VATS) and robot-assisted minimally invasive esophagectomy (RAMIE), have been introduced to improve postoperative outcomes. This study aimed to assess the impact of surgical approach on postoperative pulmonary function recovery.

**Methods:**

A retrospective analysis was conducted on patients who underwent esophagectomy between 2006 and 2023. Patients were categorized into three groups: open esophagectomy (OE), VATS, and RAMIE. Pulmonary function was assessed using vital capacity (VC) and forced expiratory volume in one second (FEV1.0) at one, three, six, and 12 months postoperatively. Multivariate logistic regression was performed to identify independent factors influencing respiratory function decline.

**Results:**

In all approaches, the VC and FEV1.0 decreased at one month postoperatively, followed by gradual recovery. Compared with OE, RAMIE showed a significantly better VC than OE at one month postoperatively (*p* = 0.04), whereas VATS showed a significantly better FEV1.0 at one month (*p* = 0.03). By 12 months, respiratory function recovery was comparable among all groups. Postoperative pulmonary complications (PPCs) were identified as a significant risk factor for long-term pulmonary function decline (*p* = 0.044).

**Conclusions:**

Minimally invasive approaches, particularly RAMIE and VATS, contribute to preserving postoperative respiratory function at one month. However, respiratory function recovery is similar across all surgical techniques at one year. Preventing PPCs is crucial for improving respiratory function outcomes. Further prospective studies are needed to validate these findings and refine surgical strategies.

**Supplementary Information:**

The online version contains supplementary material available at https://doi.org/10.1007/s10388-026-01241-z.

## Introduction

Esophagectomy for patients with esophageal cancer is a highly invasive surgical procedure that causes significant mechanical and functional changes in the thoracic wall and pulmonary capacity. These changes often lead to postoperative pulmonary dysfunction, including a decline in vital capacity (VC) and forced expiratory volume in one second (FEV1.0) [1, 2]. Previous studies on thoracic surgery have reported that the VC and FEV1.0 tend to recover to some extent within the first year after surgery [3–8]. However, the pattern of recovery may differ depending on the surgical approach.

Minimally invasive surgery has been developed to reduce surgical trauma and improve postoperative outcomes [7, 8]. Compared with conventional open esophagectomy (OE), video-assisted thoracoscopic surgery (VATS) has been reported to facilitate early postoperative respiratory function recovery. More recently, robot-assisted minimally invasive esophagectomy (RAMIE) has emerged as an advanced alternative surgical technique. RAMIE has been suggested to yield superior outcomes in terms of operative time, postoperative pain, and perioperative complications [9, 10]. However, a comparison of OE, VATS, and RAMIE with respect to respiratory function has not yet been reported. The objective of this study was to compare the effects of OE, VATS, and RAMIE on postoperative respiratory function recovery in patients who underwent esophagectomy for esophageal cancer.

## Methods

### Patients

**Fig. 1:**
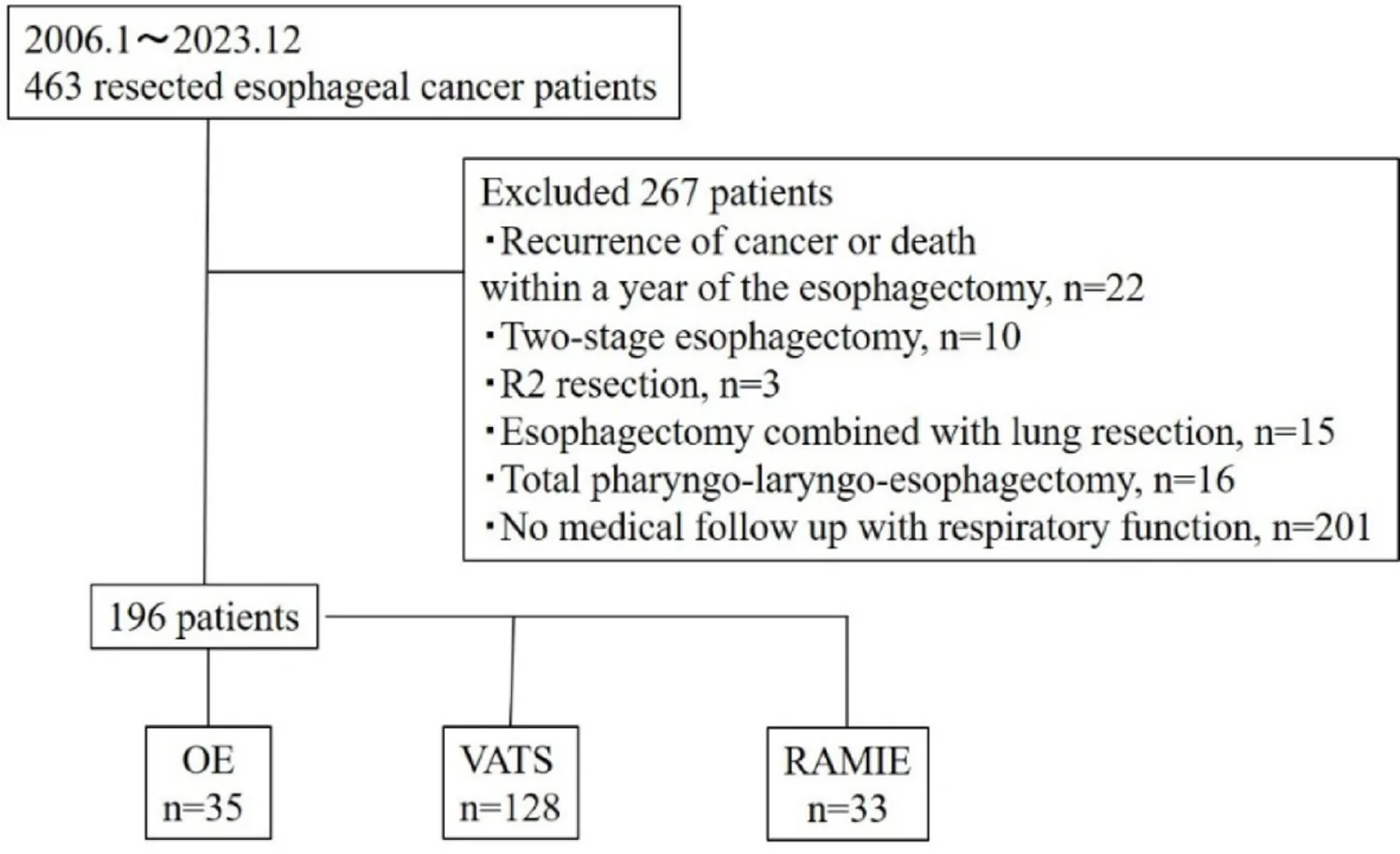
Flow diagram of the study patients. *OE* open esophagectomy, *VATS* video-assisted thoracic surgery, *RAMIE* Robot-assisted minimally invasive esophagectomy

Between January 2006 and December 2023, 463 patients underwent esophagectomy with lymph node dissection and reconstruction for esophageal cancer at Nagoya University Hospital. 267 patients who met the following criteria were excluded: recurrence of cancer or death within one year of esophagectomy, two-stage esophagectomy, noncurative resection, esophagectomy combined with lung resection, total pharyngo-laryngo-esophagectomy, and insufficient follow-up data on respiratory function. The remaining 196 patients composed the present study population. To evaluate differences in respiratory function recovery among surgical approaches, the 196 patients were categorized into three groups: OE, VATS, and RAMIE (Fig. 1). Patients treated between 2006 and 2023 were included because this was the earliest year from which complete clinical and respiratory function data were available. In addition, earlier cases were necessary to secure an adequate number of patients undergoing OE, since surgical practice at our institution transitioned from OE to VATS and subsequently to RAMIE during this period. Annual case numbers by surgical approach are presented in Supplementary Fig. 1. This study was approved by the Ethics Committee of Nagoya University Hospital (approval no. 2017-0475) and was conducted in accordance with the Declaration of Helsinki. The requirement for informed consent was waived owing to the retrospective nature of the study.

### Surgical procedures

All patients underwent esophagectomy with systematic mediastinal lymphadenectomy, such as three- or two-field lymph node dissection. The abdominal phase of the procedure was performed through a conventional open laparotomy. OE was performed by a muscle-sparing thoracotomy [11]. VATS was introduced at our institution in 2013 and gradually replaced OE. RAMIE was introduced in 2018, and its use has been increasing in recent years. Postoperative complications during hospitalization after esophagectomy were categorized as Grade II or above using the Clavien–Dindo (C-D) classification system [12].

### Respiratory function tests

Respiratory function tests were performed preoperatively and at one, three, six, and 12 months postoperatively, which was completed in the 178 study patients. The change rates of VC (CRV) and FEV1.0 (CRF) were evaluated using the following formulas:

$$ \:{\mathrm{CRV}}\:{\mathrm{or}}\:{\mathrm{CRF}} = \frac{{{\mathrm{p}}ostoperative\:VC\:or\:FEV1.0}}{{{\mathrm{p}}reoperative\:VC\:or\:FEV1.0}} \times 100 $$

To evaluate the effects of the surgical approach on the decrease in VC and FEV1.0, baseline values were determined using the average measurements from all patients. For short-term respiratory function, the reference values were set at one month postoperatively (CRV: 80%, CRF: 85%). For long-term respiratory function, the reference values were set at 12 months postoperatively (CRV: 90%, CRF: 96%). Multivariate analysis was performed for the CRV and CRF at one month and 12 months postoperatively.

### Statistical analysis

All the statistical analyses were conducted using SPSS version 29.0 for Windows (IBM Inc., Chicago, IL, USA). Continuous variables are presented as median (range), and categorical variables are presented as frequency (percentage). A p value of < 0.05 was considered to indicate statistical significance. The Wilcoxon signed-rank test was used to assess the statistical significance of changes in postoperative respiratory function (RF) over time. Categorical variables, including clinical and pathological characteristics among the three groups and preoperative RF differences, were analyzed using the chi-square test or Fisher’s exact test. Normally distributed continuous variables were analyzed using analysis of variance (ANOVA), whereas non-normally distributed continuous variables were analyzed using the Kruskal–Wallis test. To investigate the impact of different surgical approaches on postoperative RF, univariate and multivariate logistic regression analyses were performed. Odds ratios (ORs) and 95% confidence intervals (CIs) were calculated. The following clinical and pathological factors were included in the univariate analysis: age, body mass index (BMI), obstructive ventilatory disorder (OVD), surgical approach (OE, VATS, or RAMIE), neoadjuvant chemotherapy (NAC), neoadjuvant chemoradiotherapy, and postoperative pulmonary complications (pneumonia, atelectasis, pleural effusion, pulmonary embolism, and respiratory failure).

As a sensitivity analysis, we assessed the trajectory of %VC and FEV1.0 overtime at 1, 3, 6, and 12 months postoperatively using linear mixed effects model. The model adjusted for age, sex, and preoperative VC or FEV1.0.

## Results

The characteristics of the patients are summarized in Table 1. There were no significant differences among the three groups in terms of age or sex. However, the %VC was significantly lower in the VATS group (OE: 114%, VATS: 105%, RAMIE: 109%, *p* = 0.007), whereas no significant difference was observed in the FEV1.0%. Tumor progression did not significantly differ among the three groups. The proportion of patients who underwent neoadjuvant chemoradiotherapy was significantly lower in the RAMIE group (OE: 31%, VATS: 13%, RAMIE: 3%, *p* = 0.002).

**Table 1:** Patients’ characteristics

Variable	OE (*n* = 35)	VATS (*n* = 128)	RAMIE (*n* = 33)	*P* value
Age (years)	63.5 (43–78)	67.5 (41–83)	67.3 (46–81)	0.09
Sex, n				0.29
Male/Female	30/5	107/21	24/9	
PS, n				0.49
0/1	27/8	110/18	30/3	
ASA, n				0.5
1/2/3	18/13/4	55/62/11	18/11/1	
BMI (kg/㎡)	20.2 (14.5–26.0)	21.8 (11.5–46.2)	20.9 (16.1–25.3)	0.06
Alb (g/dl)	3.9 (3.1–4.5)	3.9 (2.5–5.1)	3.9 (3.2–4.7)	0.88
%VC, (%)	114 (78.6–166)	105 (68–150)	109(82–158)	0.007
FEV1.0%, (%)	73.7 (56–92.2)	73.7 (32.6–97.8)	74.4 (63.6–93.5)	0.92
OVD, n	14 (40%)	37 (29%)	10 (30%)	0.47
RVD, n	1 (3%)	3 (2%)	0 (0%)	0.65
Smoking history, n	31 (89%)	97 (76%)	26 (79%)	0.26
cStage (UICC 8th), n				
I/II/III/IV	11/8/13/3	48/32/42/6	14/6/11/2	0.9
Preoperative treatment, n				
NAC/NACRT/None	8/11/16	67/16/45	19/1/13	0.002

*PS* performance status, *ASA* American Society of Anesthesiologists, *BMI* body mass index, *%VC* percentage vital capacity, *FEV1.0%* Forced expiratory in 1 s as percent of forced vital capacity, *OVD* obstructive ventilatory defects, *RVD* restrictive ventilatory defects, UICC Union for International Cancer Control, *NAC* neoadjuvant chemotherapy, *NACRT* neoadjuvant chemoradiotherapy

The perioperative factors are presented in Table 2. A significant difference was observed in the presence or absence of thoracic duct resection (OE: 46%, VATS: 23%, RAMIE: 3%, *p* < 0.001). There was no significant difference in the operative time among the three groups. In contrast, blood loss was significantly greater in the OE group than in the other groups (median, OE: 1061 ml [315–3494 ml], VATS: 459 ml [60–2140 ml], RAMIE: 304 ml [47–1297 ml], *p* < 0.001). The incidence of complications classified as Clavien‒Dindo grade 2 or higher did not significantly differ among the three groups. The length of hospital stay was significantly shorter in the RAMIE group (median, OE: 28 days [15–94 days], VATS: 30 days [13–177 days], RAMIE: 20 days [11–75 days], *p* < 0.001).

**Table 2:** Perioperative factors

Variable	OE (*n* = 35)	VATS (*n* = 128)	RAMIE (*n* = 33)	*P* value
Field of lymphadenectomy, n				0.44
2/3	5/30	23/105	3/30	
Thoracic duct resection, n (%)	16 (46%)	30 (23%)	1 (3%)	< 0.001
Reconstruction route, n (%)				0.02
Posterior mediastinal	17 (49%)	75 (59%)	27 (82%)	
Subcutaneous	7 (20%)	28 (22%)	5 (15%)	
Retrosternal	11 (31%)	25 (19%)	1 (3%)	
Organ used for reconstruction, n (%)				0.95
Gastric tube	28 (80%)	104 (81%)	28 (85%)	
Jejunum	7 (20%)	22 (17%)	5 (15%)	
Colon	0 (0%)	2 (2%)	0 (0%)	
Blood loss, median, ml	1061 (315–3494)	459 (60–2104)	304 (47–1297)	< 0.001
Operative time, median, min	571 (337–963)	576 (321–1040)	612 (480–872)	0.76
All complication^✳^, n (%)	20 (57%)	70 (55%)	16 (48%)	0.75
Postoperative pulmonary complication, n (%)	11 (48%)	44 (34%)	9 (27%)	0.73
Postoperative RLN paralysis, n (%)	12 (34%)	48 (38%)	11 (33%)	0.88
Anastomotic leakage, n (%)	2 (6%)	29 (23%)	5 (15%)	0.063
Length of hospital stay after operation, median, day	28 (15–94)	30 (13–177)	20 (11–75)	< 0.001
Adjuvant chemotherapy, n (%)	8 (23%)	12 (9.4%)	5 (15%)	0.46
Pneumoniae in 1 year, n (%)	2 (6%)	7 (5%)	1 (3%)	0.84

*RLN* recurrent laryngeal nerve; ✳ Clavien Dindo Classification ≧ 2

**Fig. 2:**
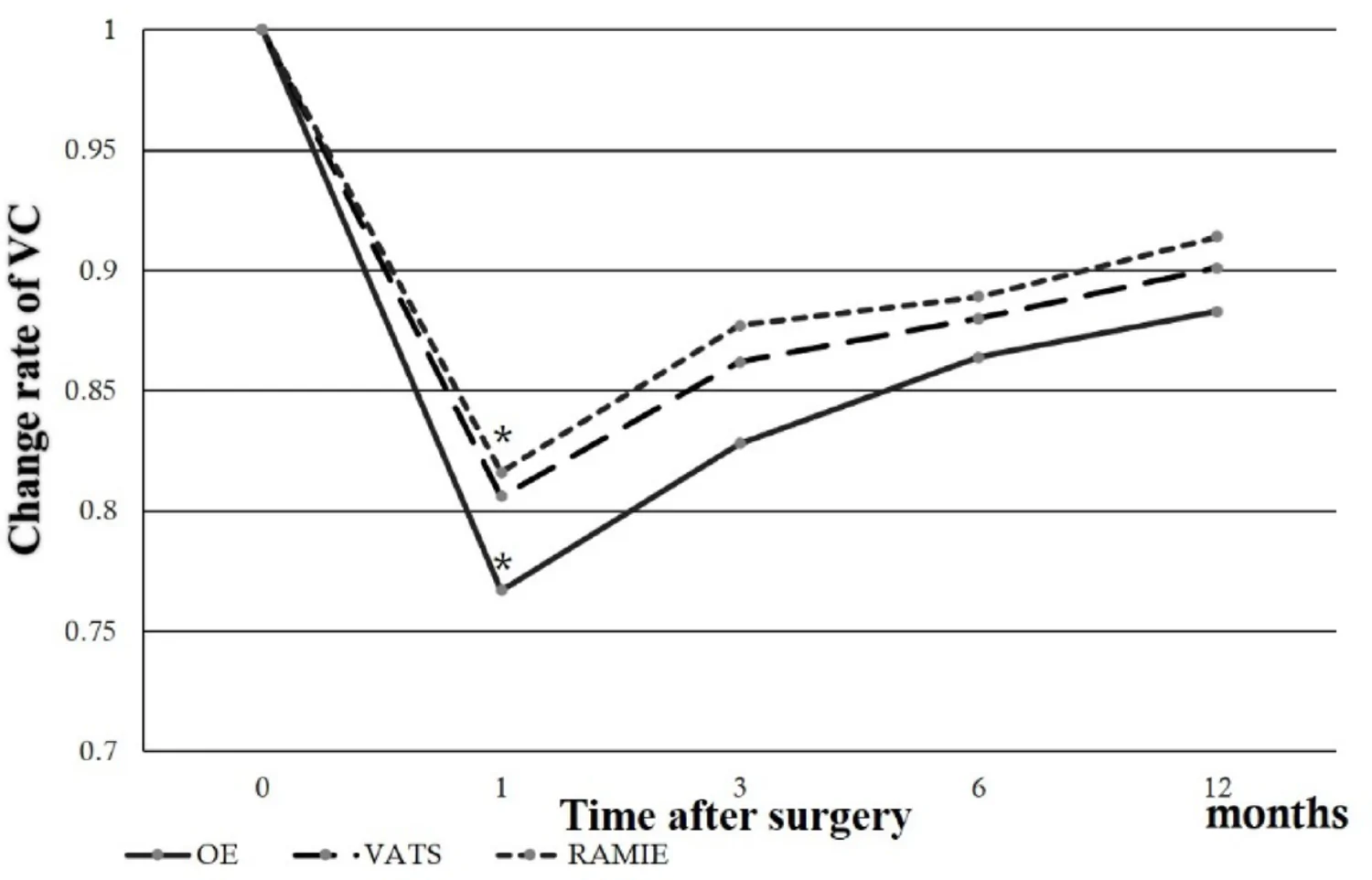
Changes in vital capacity (VC) over 12 months after esophagectomy in three surgical groups: open esophagectomy (OE), video-assisted thoracoscopic surgery (VATS), and robot-assisted minimally invasive esophagectomy (RAMIE). This figure shows the temporal changes in the change rate of VC (CRV). At one month, CRV was lowest in the OE group (76.9%) and highest in the RAMIE group (83.1%). Values are expressed as percentages of preoperative VC. Asterisks (*) indicate statistically significant differences compared with OE at the corresponding time point (*p < 0.05). Detailed values are provided in Supplementary Table 1

The changes in VC over one year among the three groups are illustrated in Fig. 2. Supplementary Table 1 A presents the detailed values for VC. All groups exhibited a decline in CRV at 1 month postoperatively (OE: 76.7%, VATS: 80.6%, RAMIE: 81.6%), especially the OE group, which tended to show a greater decline among the three groups. However, by 12 months postoperatively, the CRV had recovered to approximately 90% of the preoperative value across all surgical approaches (OE: 88.3%, VATS: 90.1%, RAMIE: 91.4%). A significant difference was observed between the RAMIE and OE groups at one month postoperatively (81.6% vs. 76.7%, *p* = 0.04). No significant differences were found at the other time points.

**Fig. 3:**
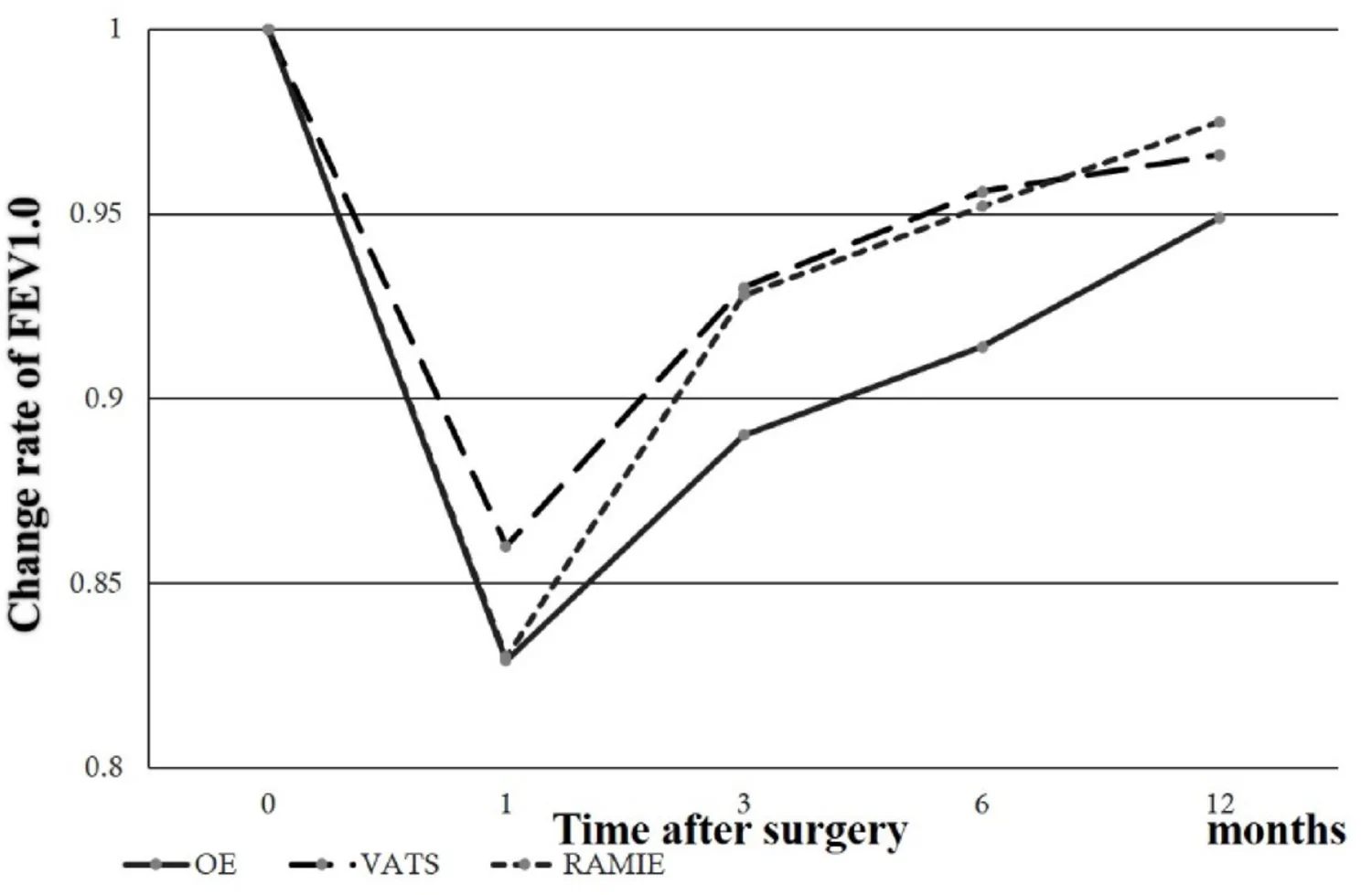
Changes in forced expiratory volume in one second (FEV1.0) over 12 months in the OE, VATS, and RAMIE groups. This figure shows the temporal changes in the change rate of FEV1.0 (CRF). At one month, CRF was slightly higher in the VATS group (85.8%) compared with OE (83.1%) and RAMIE (84.0%). Values are expressed as percentages of preoperative FEV1.0. No statistically significant differences were observed between groups at any time point. Detailed values are provided in Supplementary Table 1

The transition in the CRF over one year is shown in Fig. 3. Supplementary Table 1B presents the detailed values for FEV1.0. Similar to the VC group, all the other groups exhibited a decrease in the CRF at one month postoperatively (OE: 82.9%, VATS: 86%, RAMIE: 83%). The CRF gradually improved over time, reaching over 95% of the preoperative value at 12 months postoperatively (OE: 94.9%, VATS: 96.6%, RAMIE: 97.5%). No significant differences were observed between the surgical approaches at any time point.

The results of the univariate logistic regression analyses are shown in Supplementary Tables 2 A and 2B. The multivariate analysis results for the CRV and CRF are shown in Tables 3 and 4. The results demonstrated that RAMIE was an independent factor for preventing CRV decline at one month (OR 0.28, *p* = 0.035), but no significant difference was observed at 12 months (OR 0.46, *p* = 0.16). At 12 months, postoperative respiratory complications were identified as an independent risk factor for CRV decline (OR 2.0, *p* = 0.044). With respect to CRF, VATS was found to be an independent factor for preventing its decline at one month postoperatively (OR 0.33, *p* = 0.026), although no significant difference was observed at 12 months (OR 2.0, *p* = 0.12). Additionally, a BMI of < 18.5 was identified as an independent risk factor for CRF decline at 12 months postoperatively (OR 3.2, *p* = 0.008). The linear mixed-effects model showed similar trends to those observed in the main analysis (Supplementary Fig. 2).

**Table 3:** Risk factors for VC deterioration after esophagectomy

VC	Multivariate		
Time (1 month later)	OR	95% CI	P value
Operative approach			
OE	1.0		
VATS	0.45	0.17–1.2	0.11
RAMIE	0.28	0.08–0.91	0.035
NAC	1.7	0.84–3.4	0.14
NACRT	1.2	0.4–3.8	0.72
OVD	0.77	0.38–1.5	0.46
BMI < 18.5	1.7	0.7–4.1	0.24
Pulmonary complication	0.86	0.42–1.7	0.67
Time (12 months later)			
Operative approach			
OE	1.0		
VATS	0.75	0.33–1.7	0.5
RAMIE	0.46	0.15–1.4	0.16
NAC	1.2	0.62–2.4	0.58
NACRT	1.9	0.74–5.1	0.18
OVD	0.67	0.34–1.3	0.24
BMI < 18.5	0.94	0.42–2.1	0.88
Pulmonary complication	2	1.0–4.0	0.044

*OE* open esophagectomy, *VATS* video-assisted thoracic surgery, *RAMIE* Robot-assisted minimally invasive esophagectomy, *OVD* obstructive ventilatory defects, *NAC* neoadjuvant chemotherapy, *NACRT* neoadjuvant chemoradiotherapy, *BMI* body mass index

**Table 4:** Risk factors for FEV1.0 deterioration after esophagectomy

FEV1.0	Multivariate		
Time (1 month later)	OR	95% CI	P value
Operative approach			
Open	1.0		
VATS	0.33	0.12–0.88	0.026
RAMIE	0.74	0.23–2.3	0.6
NAC	1.3	0.66–2.7	0.43
NACRT	1.7	0.56–5.4	0.34
OVD	0.45	0.21–0.93	0.03
BMI < 18.5	0.78	0.31–2.0	0.6
Pulmonary complication	0.65	0.31–1.3	0.25
Time (12 months later)			
Operative approach			
Open	1.0		
VATS	2	0.83–4.9	0.12
RAMIE	2.2	0.7–6.7	0.18
NAC	0.65	0.33–1.3	0.21
NACRT	2.2	0.82–5.9	0.12
OVD	0.67	0.34–1.3	0.25
BMI < 18.5	3.2	1.4–7.5	0.008
Pulmonary complication	1.6	0.8–3.2	0.18

*OE* open esophagectomy, *VATS* video-assisted thoracic surgery, *RAMIE* Robot-assisted minimally invasive esophagectomy, *OVD* obstructive ventilatory defects, *NAC* neoadjuvant chemotherapy, *NACRT* neoadjuvant chemoradiotherapy, *BMI* body mass index

## Discussion

Our findings demonstrated that, compared with OE, RAMIE significantly reduced the postoperative decline in VC at one month. Similarly, VATS significantly reduced the decrease in FEV1.0 at the same time point. However, no significant differences in respiratory function recovery were observed between the RAMIE group and the VATS group at any time point. These findings suggest that both RAMIE and VATS may help mitigate early postoperative declines in respiratory function. Several factors may explain the early postoperative advantage of RAMIE and VATS over OE. Compared with OE, both minimally invasive techniques are associated with reduced chest wall trauma, lower postoperative pain, and minimal pleural adhesions. According to existing report, RAMIE has been reported to be superior in reducing postoperative pain compared with open thoracotomy [13]. Previous studies have suggested that a reduction in postoperative pain and preservation of accessory respiratory muscles significantly impact respiratory function recovery. For example, comparisons between muscle-sparing thoracotomy and posterolateral thoracotomy have indicated that muscle preservation can alleviate postoperative chest pain and promote respiratory function recovery [11]. Previous report has also suggested that pleural adhesions, such as those resulting from pleurodesis, can lead to a reduction in VC [14]. These findings support the idea that OE may also be associated with an early postoperative decline in VC.

Previous studies have reported that at one year postoperatively, VATS was superior to open esophagectomy in preserving the VC or FEV1.0 [7, 8]. On the other hand, in our study the VC and FEV1.0 significantly improved one year postoperatively, reaching approximately 90% and 95% of their preoperative values, respectively, regardless of the surgical approach. From a long-term perspective, the impact of the surgical approach on respiratory function is limited. The primary determinants of long-term respiratory function recovery are likely associated with wound healing and the resolution of postoperative pain. The process of wound healing is generally thought to be complete over the course of several months to a year [15]. Although one report suggested that thoracoscopic surgery may reduce postoperative pain in the early period after surgery, no difference in pain scores was observed between thoracoscopic surgery and thoracotomy beyond one month [16]. From this perspective, it is understandable that no differences in respiratory function were observed among surgical approaches at one year postoperatively.

Our study also identified postoperative pulmonary complications (PPCs) as an independent risk factor for postoperative respiratory function decline. Postoperative pneumonia or atelectasis may lead to pleural adhesions and fibrotic changes, thereby limiting lung expansion and contributing to the restrictive impairment reflected in reduced VC. A thoracic surgery report identified PPCs as a key risk factor for greater VC decline at one year, supporting our findings [17]. PPCs following esophagectomy have been reported in approximately 30% of cases after open thoracotomy and approximately 16% after thoracoscopic surgery [18]. Although no statistically significant differences were observed in our study, the incidence of pulmonary complications tended to be lower in the VATS and RAMIE groups than in the OE group. Owing to its higher incidence of postoperative pulmonary complications, OE may result in greater impairment of respiratory function.

This study has several limitations. First, potential selection bias should be acknowledged, as a substantial proportion of patients were excluded due to missing postoperative pulmonary function data. Many of the excluded cases dated back to earlier periods, when postoperative respiratory function testing was not routinely performed, and open esophagectomy was more commonly performed. Consequently, more than half of the excluded cases underwent open esophagectomy and tended to have poorer preoperative conditions, including lower %VC and BMI. As a result, the postoperative decline in respiratory function in the OE group may have been underestimated. Second, the surgical approaches in this study varied in adoption timing and technical maturity: OE cases were mostly from the late phase, VATS from early to mature phases, and RAMIE mainly from the early phase. This temporal variation may *have led* to background heterogeneity, potentially affecting the outcomes. Third, the OE and RAMIE groups remained relatively small compared with the VATS group. In addition, the adoption of muscle-sparing thoracotomy in the OE group may have contributed to relatively favorable outcomes. These factors represent potential confounding influences, and results related to OE and RAMIE should be interpreted with caution. Fourth, unmeasured confounders such as surgical experience, year of surgery, and institutional learning curves may have influenced both treatment selection and outcomes due to the long study period. Fifth, although we adjusted for potential confounders using multivariate analysis and confirmed the consistency of our findings through sensitivity analysis using a linear mixed model, the substantial imbalance in baseline characteristics across surgical approaches-especially between OE and the others- suggests the potential influence of residual confounding. This limitation was also discussed in consultation with a statistician. Further prospective, well-designed controlled trial is needed to confirm these results and refine surgical strategies for optimizing postoperative respiratory function.

## Conclusion

This study compared the effects of OE, VATS, and RAMIE on postoperative respiratory function. The results showed that VATS and RAMIE helped mitigate the early postoperative decline in respiratory function, but all surgical approaches achieved similar recoveries by 12 months. RAMIE was identified as an independent factor in reducing early VC decline.

## Supplementary Information

Below is the link to the electronic supplementary material.

Supplementary Material 1
