# Supplementary material for: Comparison of Respiratory function recovery after esophagectomy: a retrospective analysis of surgical approaches
Source: Esophagus. 2026 Sep 20;23(4):905–12. doi: 10.1007/s10388-026-01241-z (PMC13723442; doi:10.1007/s10388-026-01241-z)
Supplement: Supplementary file 1 [file 10388_2026_1241_MOESM1_ESM.docx]

**Supplementary Table 1. Mean VC and FEV1.0 values by surgical group and timepoint.**

A. **Vital Capacity (VC, L) and Change rate of VC (CRV, %)**

| **Time point** | OE VC/ CRV | VATS VC/ CRV | RAMIE VC/ CRV |
| --- | --- | --- | --- |
| Pre-operation | 3.75/ - | 3.6/ - | 3.67/ - |
| 1 month | 2.89/ 76.7 | 2.91/ 80.6 | 3.03/ 81.6 |
| 3 months | 3.2/ 82.8 | 3.12/ 86.2 | 3.11/ 87.7 |
| 6 months | 3.31/ 86.4 | 3.17/ 88 | 3.31/ 88.9 |
| 12 months | 3.32/ 88.3 | 3.25/ 90.1 | 3.4/ 91.4 |

B. **Forced Expiratory Volume in 1 second (FEV1.0) and Change rate of FEV1.0**

**(CRF)**

| **Time point** | OE FEV1.0/ CRF | VATS FEV1.0/ CRF | RAMIE FEV1.0/ CRF |
| --- | --- | --- | --- |
| Pre-operation | 2.74/ - | 2.6/ - | 2.66/ - |
| 1 month | 2.22/ 82.9 | 2.23/ 86 | 2.23/ 83 |
| 3 months | 2.51/ 89.0 | 2.47/ 93 | 2.41/ 92.8 |
| 6 months | 2.55/ 91.4 | 2.49/ 95.6 | 2.58/ 95.2 |
| 12 months | 2.57/ 94.9 | 2.52/ 96.6 | 2.61/ 97.5 |

ALL values are presented as mean values (L or %).

**Supplementary Table 2.
A. Univariate logistic regression analysis for postoperative VC decline after esophagectomy.**

| **VC** | Univariate | | |
| --- | --- | --- | --- |
| Time (1 month later) | OR | 95% CI | P value |
| Operative approach |  |  |  |
| OE | 1.0 |  |  |
| VATS | 0.45 | 0.19- 1.1 | 0.07 |
| RAMIE | 0.27 | 0.09- 0.79 | 0.02 |
| NAC | 1.8 | 0.94- 3.3 | 0.08 |
| NACRT | 2.4 | 0.89- 6.4 | 0.08 |
| OVD | 0.82 | 0.43- 1.6 | 0.54 |
| BMI<18.5 | 2.2 | 0.97- 4.9 | 0.06 |
| Pulmonary complication | 0.96 | 0.5- 1.8 | 0.89 |
|  |  |  |  |
|  |  |  |  |
| Time (12 months later) |  |  |  |
| Operative approach |  |  |  |
| OE | 1.0 |  |  |
| VATS | 0.74 | 0.35- 1.6 | 0.43 |
| RAMIE | 0.4 | 0.14- 1.1 | 0.08 |
| NAC | 1.4 | 0.76- 2.6 | 0.28 |
| NACRT | 2.5 | 1.1- 6.1 | 0.04 |
| OVD | 0.74 | 0.39- 1.4 | 0.36 |
| BMI<18.5 | 1.4 | 0.68- 2.9 | 0.35 |
| Pulmonary complication | 2 | 1.1- 3.8 | 0.03 |

*OE* open esophagectomy, *VATS* video-assisted thoracic surgery, *RAMIE* Robot-assisted minimally invasive esophagectomy, *OVD* obstructive ventilatory defects, *NAC* neoadjuvant chemotherapy, *NACRT* neoadjuvant chemoradiotherapy, *BMI* body mass index

**B. Univariate logistic regression analysis for postoperative FEV1.0 decline after esophagectomy.**

| **FEV1.0** | Univariate | | |
| --- | --- | --- | --- |
| Time (1 month later) | OR | 95% CI | P value |
| Operative approach |  |  |  |
| Open | 1.0 |  |  |
| VATS | 0.39 | 0.17- 0.92 | 0.03 |
| RAMIE | 0.78 | 0.28- 2.2 | 0.64 |
| NAC | 1.2 | 0.65- 2.2 | 0.56 |
| NACRT | 2.0 | 0.75- 5.2 | 0.17 |
| OVD | 0.52 | 0.27- 1.0 | 0.05 |
| BMI<18.5 | 1.0 | 0.46- 2.2 | 0.97 |
| Pulmonary complication | 0.58 | 0.3- 1.1 | 0.11 |
|  |  |  |  |
|  |  |  |  |
| Time (12 months later) |  |  |  |
| Operative approach |  |  |  |
| Open | 1.0 |  |  |
| VATS | 1.4 | 0.65- 3.0 | 0.39 |
| RAMIE | 1.3 | 0.48- 3.6 | 0.61 |
| NAC | 0.83 | 0.45- 1.5 | 0.53 |
| NACRT | 1.7 | 0.73- 3.9 | 0.22 |
| OVD | 0.6 | 0.32- 1.1 | 0.12 |
| BMI<18.5 | 3.3 | 1.5- 7.2 | 0.003 |
| Pulmonary complication | 2.0 | 1.0- 3.7 | 0.04 |

*OE* open esophagectomy, *VATS* video-assisted thoracic surgery, *RAMIE* Robot-assisted minimally invasive esophagectomy, *OVD* obstructive ventilatory defects, *NAC* neoadjuvant chemotherapy, *NACRT* neoadjuvant chemoradiotherapy, *BMI* body mass index

**Supplementary Figure 1. Annual number of esophagectomy cases by surgical approach (OE, VATS, RAMIE) between 2006 and 2023."**

The transition of surgical approach adoption over time is shown. VATS gradually replaced OE, and RAMIE was introduced in later years.

**Supplementary Figure 2.**

**A. Trends in VC (L) evaluated by linear mixed-effects models.**

B. **Trends in FEV1.0 (L) evaluated by linear mixed-effects models.**

Asterisks indicate significant differences between OE and RAMIE groups at specific time points. (p=0.021)

Line graphs show the estimated marginal means of VC and FEV1.0 over 12 months, adjusted for age, sex, and preoperative values.
At 1 month postoperatively, RAMIE demonstrated significantly less decline in VC compared with OE (p = 0.021).
For FEV1.0, although the difference between VATS and OE was not statistically significant at 1 month (p = 0.054), a favorable trend toward better recovery was observed in the VATS group.
